# Cerebrovascular diseases in ADHD patients with metabolic comorbidities: a retrospective cohort study

**DOI:** 10.1186/s12888-025-07246-9

**Published:** 2025-08-20

**Authors:** Sarah Krieg, Andreas Krieg, Karel Kostev

**Affiliations:** 1 University Clinic for People with Neurodevelopmental Disorders, Mara Hospital, Medical School and University Medical Center OWL, Bielefeld University, Bielefeld, 33617 Germany; 2 Department of General and Visceral Surgery, Thoracic Surgery and Proctology, Medical Campus OWL, University Hospital Herford, Ruhr University Bochum, Herford, 32049 Germany; 3 Epidemiology, IQVIA, Frankfurt, Germany; 4 University Clinic, Philipps-University, Marburg, Germany

**Keywords:** ADHD, Cerebrovascular risk, Ischemic stroke, Transient ischemic attack, Metabolic comorbidities, Cardiovascular risk, Diabetes, Dyslipidemia, Long-term risk

## Abstract

**Background:**

ADHD is associated with several health outcomes, but its long-term cerebrovascular associations, particularly in adults with metabolic conditions, remain poorly understood.

**Methods:**

A retrospective cohort analysis was performed using the Disease Analyzer database (IQVIA) and included adults with ADHD which were compared to a propensity score matched non-ADHD cohort. The 10-year incidence of acute ischemic stroke (AIS) or transient ischemic attack (TIA) was analyzed using Kaplan-Meier curves and univariable conditional Cox regression models with hazard ratios (HRs) and 99% confidence intervals (CIs).

**Results:**

Among 8,943 ADHD patients and 44,660 controls, 1.7% of ADHD patients and 1.2% of controls experienced AIS or TIA (log-rank test, p = 0.006). In the regression analysis, there was a significant association between ADHD and subsequent TIA or AIS in the total population (HR: 1.69; 99% CI:1.13-2.51). This association was stronger and significant in subgroups of patients aged >45 years (HR: 2.34; 99% CI: 1.43-3.81) and those with obesity (HR: 1.86; 99% CI: 1.22-2.83), hypertension (HR: 2.18; 99% CI: 1.19-3.97), or dyslipidemia (HR: 2.89; 99% CI: 1.48-5.52).

**Conclusion:**

ADHD in adults, particularly those over 45 years of age and those with metabolic comorbidities, is associated with an increased risk of cerebrovascular disease. Further research is needed to confirm these associations and to explore the underlying mechanisms.

## Introduction

Attention-deficit/hyperactivity disorder (ADHD) is a neuropsychiatric disorder characterized by persistent symptoms of inattention, hyperactivity, and impulsivity [1]. It typically manifests in childhood but can persist into adulthood [1, 2]. The global prevalence of ADHD is estimated to be 5–7% in children and 2–5% in adults [2].

The standard pharmacological treatment for ADHD primarily involves central nervous system (CNS) stimulants, such as methylphenidate and amphetamines [3]. These stimulants modulate dopamine and noradrenaline levels by inhibiting their reuptake into presynaptic neurons and increasing their release [3]. This process prolongs the availability of these neurotransmitters at the synaptic cleft and improves neuronal signaling, clinically leading to enhanced attention regulation and impulse control [3]. However, evidence from clinical trials suggests that CNS stimulants may be associated with adverse cardiovascular effects, including increased blood pressure and heart rate [4, 5]. Using data from a self-controlled case-series analysis, Shin et al. investigated the association between methylphenidate treatment and cardiovascular outcomes in children and adolescents with ADHD using the South Korean national health insurance database (2008–2011) [5]. A significantly increased incidence of arrhythmias was observed across all treatment periods (incidence rate 1.61, 95% CI 1.48–1.74), with the highest risk identified in children with congenital heart disease. Although no significantly increased incidence of myocardial infarction was found for all exposure periods (1.33; 0.90–1.98), the risk was elevated during the early weeks of treatment [5].

In addition, adults with ADHD are also more likely to engage in lifestyle behaviors that negatively impact cardiovascular health, including obesity, tobacco use, and substance abuse, all of which are independently associated with an increased incidence of cardiovascular disease [6, 7]. Given the combined impact of these risk factors and the potential cardiovascular effects of long-term stimulant use, concerns have emerged regarding an increased probability of major adverse cardiovascular events (MACE) in this patient population [4, 8].

Acute ischemic stroke (AIS) and transient ischemic attack (TIA) are among the most common cerebrovascular conditions and represent major causes of morbidity and mortality worldwide [9–11]. Notably, cerebrovascular and cardiovascular diseases share common risk factors, including hypertension, diabetes, and dyslipidemia, highlighting their interconnected pathophysiology and the importance of a comprehensive cardiovascular risk assessment [9–11]. Further risk factors for AIS are atrial fibrillation [12], and depression [13]. Previous research reported sex-differences in the association between risk factors and stroke [14].

Despite prior research indicating an increased incidence of cardiovascular disease in patients with ADHD, particularly in relation to pharmacotherapy [15, 16], systematic investigations explicitly examining the association between ADHD and MACE remain limited. Recent studies have further advanced this research area by exploring the link between ADHD, ADHD medication, and cerebrovascular disease [17]. However, significant gaps remain in our understanding of how ADHD itself, independent of pharmacotherapy, contributes to cerebrovascular risk. Furthermore, many prior studies have focused on pediatric and adolescent populations or have not comprehensively accounted for metabolic and age-related risk factors in adults with ADHD [18]. Since AIS and TIA occur predominantly in older and multimorbid populations [11], it is crucial to investigate the impact of ADHD in these specific subgroups.

This study aims to provide new insights into the incidence of AIS and TIA in adults with ADHD, with a particular focus on age-stratified and metabolically comorbid subpopulations. By addressing this research gap, the study seeks to contribute to the growing body of evidence and provide a foundation for improved risk stratification and prevention strategies for cerebrovascular diseases in individuals with ADHD.

## Methods

### Database

This retrospective cohort study was based on data from the Disease Analyzer database (IQVIA), which contains drug prescriptions, diagnoses, and basic medical and demographic data obtained directly and in anonymous format from computer systems used in the practices of general practitioners and specialists [19]. The database covers approximately 3000 office-based physicians in Germany. It has previously been shown that the panel of practices included in the Disease Analyzer database is representative of general and specialized practices in Germany [19]. Finally, this database has already been used in previous studies focusing on stroke and TIA [20, 21].

### Study population

#### Inclusion criteria

**Fig. 1:**
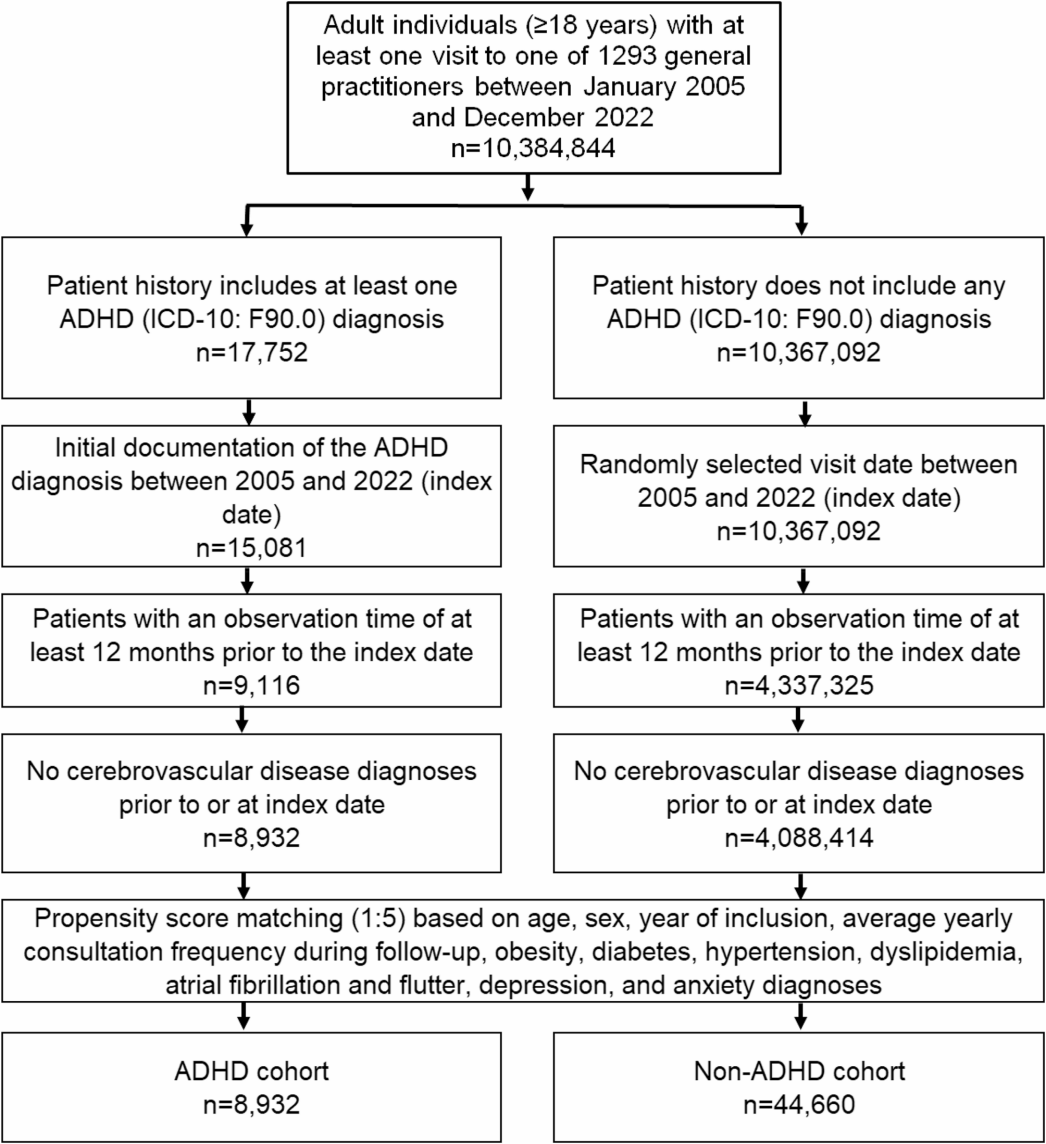
Selection of study patients

This study included adult patients (≥ 18 years at index date) with an initial diagnosis documentation of ADHD (ICD-10: F90.0) in 1,293 general practices in Germany between January 2005 and December 2022 (index date; Fig. 1). Only patients with an observation time of at least 12 months prior to the index date were included to access the co-diagnoses documented within 12 months prior to the index date.

After applying similar inclusion criteria, individuals without ADHD were matched to ADHD patients using nearest neighbor propensity score matching (5: 1) based on age, sex, year of inclusion, average yearly consultation frequency during follow-up, and co-diagnoses of obesity (ICD-10: E66), diabetes mellitus (ICD-10: E10-E14), hypertension (ICD-10: I10), dyslipidemia (ICD-10: E78), atrial fibrillation and flutter (ICD10: I48), and depression or anxiety (ICD-10: F32, F33, F41) documented within 12 months prior to or on index date. Obesity, diabetes mellitus, hypertension, dyslipidemia, atrial fibrillation, and psychiatric diseases (depression, anxiety) were included as they are risk factors for cerebrovascular diseases. Yearly consultation frequency was considered because ADHD patients visit physicians more often, which may increase the likelihood of documenting additional diagnoses. Higher age and male sex are both associated with cerebrovascular diseases, making their inclusion essential.

For the non-ADHD cohort, the index date was that of a randomly selected visit between January 2005 and December 2022 (Fig. 1). Standardized mean difference (SMD) was used to examine the balance of covariate distribution between cohorts. In this study, a SMD of less than 0.1 was allowed, indicating that adequate covariate balance has been achieved.

For a sensitivity analysis, we conducted the same nearest neighbor propensity score matching 1:1 (instead of 5:1).

#### Exclusion criteria

Patients with a diagnosis of cerebrovascular diseases, including stroke and TIA (ICD-10: I60-I69, G45.0) prior to or on index date were excluded to assess the incidence of these conditions as first-time occurrences.

### Study outcomes

The outcomes of the study were the initial diagnoses of TIA (ICD-10: G45) or AIS (ICD-10: I63, I64) in the up to 10 years following the index date as function of ADHD.

### Statistical analyses

The 10-year cumulative incidence of TIA and AIS was studied with Kaplan-Meier curves. Finally, an univariable conditional Cox regression analysis was conducted to assess the association between ADHD and TIA or AIS. These models were conducted separately for three age groups, female and male individuals, as well as individuals with obesity, diabetes, stroke, and dyslipidemia. As a sensitivity analysis, Cox regression model was repeated based on 1:1 the matched cohorts. Results of the Cox regression model have been displayed as hazard ratios (HRs) and 99% confidence intervals (CIs). P-value of < 0.01 was considered statistically significant due to multiple comparisons (five models, Bonferroni correction). Analyses were conducted using SAS version 9.4 (SAS Institute, Cary, USA).

## Results

### Baseline characteristics of the study sample

The present study included 8,943 individuals with and 44,660 without ADHD. The baseline characteristics of study patients are displayed in Table 1. Mean age was 30.1 (SD: 13.2) years, 34.9% were male. Patients visited physicians in average 5.2 times per year during the follow-up. Predefined comorbidities were not frequent (7.2% with obesity, 10.5% with hypertension, and 3.9% with diabetes, 7.7% with dyslipidemia).

**Table 1:** Baseline characteristics of the study sample (after 1:5 propensity score matching)

Variable	Proportion among patients with ADHD (*N*, %) *N* = 8,932	Proportion among patients without ADHD (*N*, %) *N* = 44,660	Standardized mean differences
Age (Mean, SD)	30.1 (13.2)	30.2 (13.2)	−0.041
Age 18–30	5,543 (62.1)	27,674 (62.0)	
Age 31–45	2,067 (23.1)	10,336 (23.1)	
Age > 45	1,322 (14.8)	6,6530 (14.9)	
Female	3,120 (34.9)	15,622 (35.0)	0.000
Male	5,812 (65.1)	29,038 (65.0)	
Number of physician visits per year during the follow-up (Mean, SD)	5.2 (4.1)	5.2 (4.1)	−0.009
Obesity	647 (7.2)	3,214 (7.2)	0.000
Diabetes mellitus	351 (3.9)	1,561 (3.5)	0.004
Hypertension	940 (10,5)	4,589 (10.3)	−0.002
Dyslipidemia	718 (8.0)	3,492 (7.8)	−0.002
Atrial fibrillation and flutter	44 (0.5)	178 (0.4)	0.000
Depression and anxiety	3,393 (38.0)	16,961 (37.9)	0.000

Proportions of patients in N, % given, unless otherwise indicated

*SD* Standard deviation

### Cumulative incidence of AIS and TIA among patients with and without ADHD

**Fig. 2:**
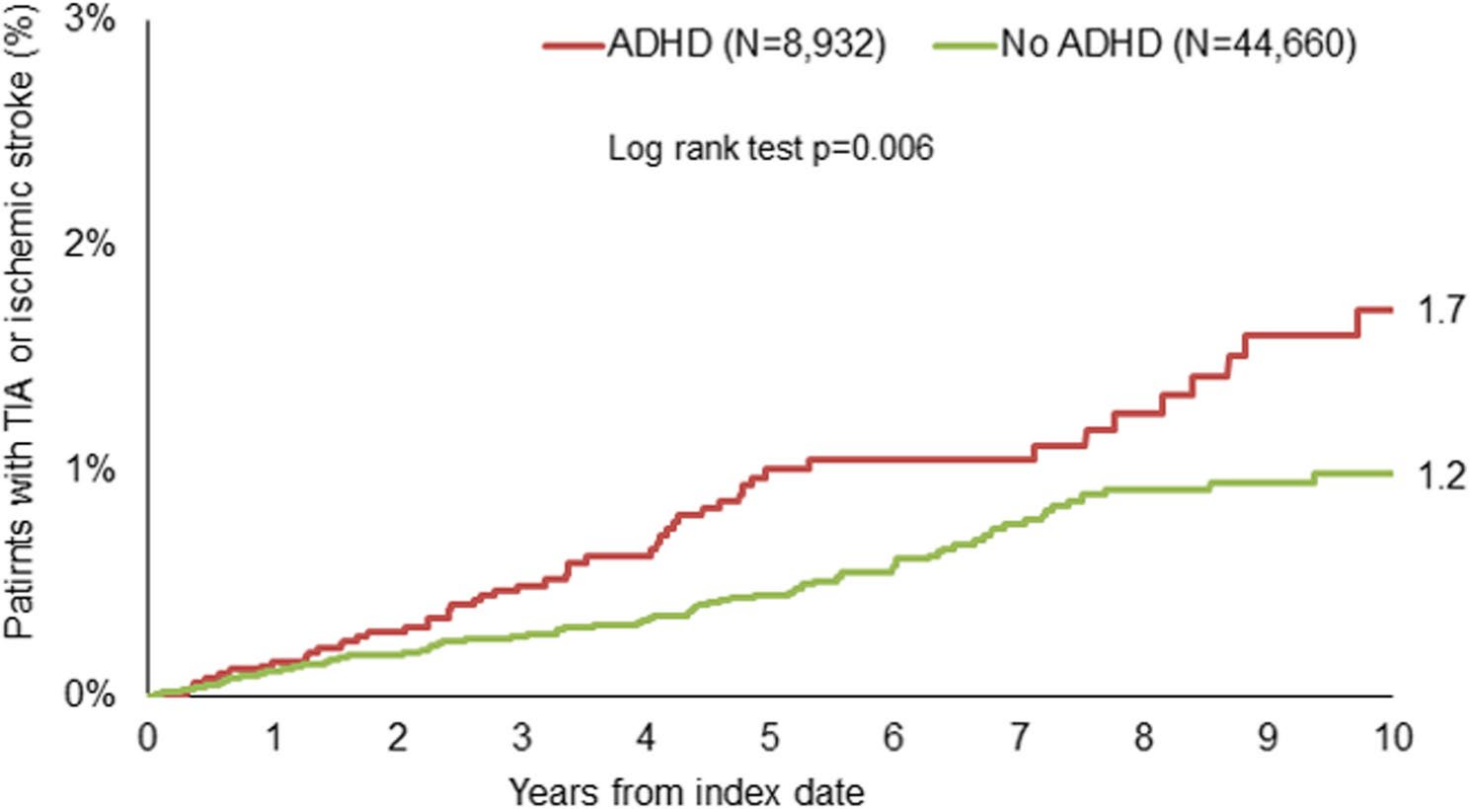
Cumulative incidence of TIA and AIS in patients with and without ADHD

After up to ten years of follow-up, 1.7% of ADHD and 1.2% of non-ADHD patients were diagnosed with TIA or AIS (*p* = 0.006, Fig. 2). Both outcome diseases were rare, but AIS was more frequent than TIA (84 vs. 124 cases). The proportional hazards assumption was met (Schoenfeld residual test; correlation = 0.019; *p* = 0.789).

### Association of ADHD with TIA or AIS

In the regression analysis, there was a significant association between ADHD and subsequent TIA or AIS in the total population (HR: 1.69; 99% CI:1.13–2.51) (Table 2).

**Table 2:** Association between ADHD and subsequent TIA and AIS diagnoses in patients followed in general practices in Germany (univariable Cox regression models)

Patient subgroup	TIA or AIS		TIA		AIS	
	HR (99% CI)	P value	HR (99% CI)	P value	HR (99% CI)	P value
Total	1.69 (1.13–2.51)	< 0.001	1.68 (0.93–3.04)	0.024	1.69 (0.99–2.90)	0.012
Age 18–30	0.84 (0.63–3.39)	0.730	0.73 (0.14–3.88)	0.624	1.08 (0.14–8.60)	0.921
Age 31–45	1.46 (0.63–3.39)	0.246	1.57 (0.52–5.39)	0.258	1.27 (0.38–4.29)	0.609
Age > 45	2.34 (1.43–3.81)	< 0.001	2.50 (1.15–5.43)	0.002	2.23 (1.19–4.19)	0.001
Female	1.62 (0.89–2.92)	0.037	1.25 (0.51–3.07)	0.515	2.02 (0.91–4.49)	0.023
Male	1.78 (1.04–3.04)	0.034	2.23 (0.99–5.04)	0.011	1.49 (0.72–3.09)	0.157
Individuals with obesity	1.86 (1.22–2.83)	< 0.001	1.34 (0.30–5.94)	0.613	0.38 (0.03–4.46)	0.351
Individuals with diabetes mellitus	2.69 (1.09–6.67)	0.005	2.03 (0.48–8.56)	0.204	3.28 (1.00-10.73)	0.009
Individuals with hypertension	2.18 (1.19–3.97)	< 0.001	2.53 (1.02–6.29)	0.009	1.94 (0.87–4.34)	0.034
Individuals with dyslipidemia	2.89 (1.48–5.52)	< 0.001	4.02 (1.46–11.05)	< 0.001	2.21 (0.91–5.39)	0.021

This association was stronger and significant in the age group > 45 years (HR 2.34; 99% CI: 1.43–3.81), individuals with obesity (HR: 1.86; 99% CI: 1.22–2.83), hypertension (HR: 2.18; 99% CI: 1.19–3.97). and dyslipidemia (HR: 2.89; 99% CI: 1.48–5.52). When regression analysis was done using 1:1 matched pairs (sensitivity analysis), te association was similar (HR: 1.61; 95% CI: 0.85–3.06) but not significant (*p* = 0.054).

When considering TIA and AIS separately, ADHD was associated with an increased risk of TIA only in individuals with dyslipidemia (HR: 4.02 99% CI: 1.45–11.05), since HR for the positive associations between ADHD and AIS was higher than 2 in the age group > 45 years, female patients, individuals with diabetes mellitus, and dyslipidemia, but p values did not reach a significance level of < 0.001 (Table 2).

## Discussion

This study provides novel insights into the association between ADHD and cerebrovascular disease (AIS; TIA), particularly in adults aged 45 and older with metabolic comorbidities. Our findings indicate that individuals with ADHD had a higher cumulative incidence of TIA and AIS compared to controls, with the association being particularly pronounced in those with obesity, hypertension, or dyslipidemia. These results align with prior research demonstrating an increased cardiovascular burden in ADHD patients, although most previous studies have focused on younger populations and the immediate cardiovascular effects of stimulant medication rather than cerebrovascular outcomes in adults [5, 17].

Zhang et al. recently conducted a large-scale case-control study using Swedish national registry data and reported an increased incidence of cardiovascular diseases, including cerebrovascular events, among ADHD patients with long-term stimulant use [17]. Their analysis demonstrated a 4% increase in cardiovascular disease incidence per additional year of ADHD medication use, with the greatest risk occurring within the first three years of treatment. Notably, prolonged medication exposure (> 5 years) was significantly associated with hypertension and arterial disease, both of which are established risk factors for cerebrovascular disease [17]. While these findings emphasize the importance of long-term cardiovascular monitoring in ADHD patients, the study did not fully differentiate between the direct impact of ADHD itself and the modifying role of pharmacotherapy [17]. Our results suggest that ADHD may be associated with cerebrovascular diseases, particularly in individuals with metabolic disorders; however, given the potential influence of unmeasured confounders, further research is needed to clarify the mechanisms underlying this association.

The potential biological pathways linking ADHD and cerebrovascular disease likely involve a complex interplay of metabolic dysregulation, vascular dysfunction, and behavioral factors [16, 22, 23]. ADHD has been associated with an increased prevalence of lifestyle-related risk factors, including smoking, physical inactivity, and unhealthy dietary habits [22, 23], all of which contribute to metabolic syndrome and cardiovascular disease [24]. Additionally, ADHD has been linked to autonomic dysregulation, which may influence vascular tone and hemodynamic stability, further exacerbating cerebrovascular risk [16].

Stimulant medications commonly prescribed for ADHD are known to increase blood pressure and heart rate. A meta-analysis by Mick et al. reported a mean increase of 5.7 beats per minute in resting heart rate and a 2.0 mmHg rise in systolic blood pressure among ADHD patients receiving stimulant therapy [4]. While these changes may appear modest, even slight elevations in blood pressure over time could contribute to cerebrovascular burden, particularly in individuals with pre-existing vascular risk factors [25]. However, Shin et al. found no significant association between ADHD medication and cerebrovascular disease in pediatric and adolescent populations, suggesting that vascular changes in adulthood, rather than stimulant-related effects alone, may play a more critical role in cerebrovascular outcomes [5].

The observed association between ADHD and cerebrovascular diseases appears to be amplified in individuals with metabolic disorders. Insulin resistance, chronic inflammation, and lipid dysregulation are key contributors to atherosclerosis and cerebrovascular pathology [26]. The metabolic score for insulin resistance (METS-IR) has been identified as a predictor of stroke risk in hypertensive patients, reinforcing the hypothesis that metabolic impairments may mediate cerebrovascular outcomes [26]. Although METS-IR was not specifically assessed in our study, the significant interaction between ADHD and metabolic comorbidities suggests that future investigations should explore the role of insulin resistance and lipid metabolism in ADHD-related cerebrovascular diseases.

Several limitations must be considered when interpreting the findings of this study. The reliance on ICD-10 coding means that conditions such as obesity, diabetes, and hypertension were identified based on general practice documentation rather than objective clinical measurements (e.g., BMI, laboratory values), which may have introduced misclassification. Additionally, diagnoses typically made in hospital, such as AIS and TIA, are often recorded retrospectively in general practice — only after this would the corresponding ICD-10 code appear in our dataset, potentially leading to underreporting of outcomes. The use of 1:5 propensity score matching improved comparability and statistical power but may have introduced residual differences between the groups, potentially affecting generalizability. Furthermore, information on ADHD symptom severity was not available, preventing an analysis of whether more severe ADHD cases were disproportionately associated with cerebrovascular diseases. Similarly, data on ADHD pharmacotherapy were inaccessible, as stimulant and non-stimulant medications are primarily prescribed by psychiatrists rather than general practitioners. This limitation precluded an assessment of the potential impact of ADHD treatment on cerebrovascular risk.

Additionally, key lifestyle factors (e.g., smoking status, physical activity, diet)—which are well-established contributors to cerebrovascular risk—were not available in the dataset, increasing the possibility of residual confounding. Given the retrospective observational design, causality cannot be established, and unmeasured confounders may have influenced the observed associations.

Despite these limitations, this study has several strengths. The large sample size enabled robust statistical analyses and improved generalizability. The application of propensity score matching accounted for key confounders such as age, sex, and metabolic comorbidities, enhancing the validity of comparisons between ADHD and non-ADHD individuals. The long follow-up period of up to ten years provided valuable insights into long-term cerebrovascular risks associated with ADHD, particularly in older adults and those with metabolic conditions. Furthermore, detailed subgroup analyses helped elucidate how metabolic comorbidities interact with ADHD in relation to cerebrovascular diseases.

## Conclusions

This study demonstrates a statistically significant association between ADHD and an increased incidence of TIA or AIS, particularly in individuals aged 45 and older and those with metabolic comorbidities such as obesity, hypertension, and dyslipidemia. These findings underscore the need for greater clinical awareness of cerebrovascular risk in ADHD patients with metabolic conditions. While causal relationships cannot be determined, the results highlight important questions about the potential role of metabolic dysregulation and cardiovascular factors in influencing cerebrovascular health in ADHD. Although residual confounding cannot be ruled out, the observed associations warrant further investigation to clarify potential underlying mechanisms. Future studies should explore the contribution of insulin resistance, chronic inflammation, and lipid metabolism to these associations. Additionally, prospective research evaluating the impact of cardiovascular risk management strategies—such as antihypertensive therapy, lipid-lowering interventions, and lifestyle modifications—on cerebrovascular outcomes in ADHD patients is needed.

From a clinical perspective, our findings suggest that ADHD patients with metabolic risk factors could benefit from closer cardiovascular monitoring and tailored prevention strategies. Given the complex interplay between ADHD, metabolic health, and cerebrovascular risk, an interdisciplinary approach involving neurologists, psychiatrists, cardiologists, and endocrinologists may be necessary to optimize risk assessment and management in this population. However, additional research is required before these findings can inform clinical guidelines. Identifying high-risk subgroups within the ADHD population may help refine prevention strategies and improve long-term cerebrovascular outcomes.

## Data Availability

No datasets were generated or analysed during the current study.

## Abbreviations

ADHD: attention deficit/hyperactivity disorder
AIS: Acute ischemic stroke
BPM: beat-per-minute
CI: Confidence interval
CNS: Central nervous system
HR: Hazard ratio
MACE: Major adverse cardiovascular event
METS-IR: Metabolic score for insulin resistance
TIA: transient ischemic attack
